# Oral Vaccination with a DNA Vaccine Encoding Capsid Protein of Duck Tembusu Virus Induces Protection Immunity

**DOI:** 10.3390/v10040180

**Published:** 2018-04-06

**Authors:** Juan Huang, Haoyue Shen, Renyong Jia, Mingshu Wang, Shun Chen, Dekang Zhu, Mafeng Liu, Xinxin Zhao, Qiao Yang, Ying Wu, Yunya Liu, Ling Zhang, Zhongqiong Yin, Bo Jing, Anchun Cheng

**Affiliations:** 1 Research Center of Avian Disease, College of Veterinary Medicine of Sichuan Agricultural University, Wenjiang District, Chengdu 611130, China; huangjuan610@163.com (J.H.); shenhaoyue0724@163.com (H.S.); mshwan@163.com (M.W.); sophia_cs@163.com (S.C.); zdk24@163.com (D.Z.); liumafengra@163.com (M.L.); xxinzhao@163.com (X.Z.); yangqiao721521@sina.com (Q.Y.); yingzi_no1@126.com (Y.W.); cqrc_jry@163.com (Y.L.); zl97451@126.com (L.Z.); 2 Institute of Preventive Veterinary Medicine, Sichuan Agricultural University, Wenjiang District, Chengdu 611130, China; 3 Key Laboratory of Animal Disease and Human Health of Sichuan Province, Wenjiang District, Chengdu 611130, China; yinzhongq@163.com (Z.Y.); jingbooo@163.com (B.J.)

**Keywords:** *Flavivirus*, duck tembusu virus, capsid protein, oral DNA vaccine, immunogenicity

## Abstract

The emergence of duck tembusu virus (DTMUV), a new member of the *Flavivirus* genus, has caused great economical loss in the poultry industry in China. Since the outbreak and spread of DTMUV is hard to control in a clinical setting, an efficient and low-cost oral delivery DNA vaccine SL7207 (pVAX1-C) based on the capsid protein of DTMUV was developed and evaluated in this study. The antigen capsid protein was expressed from the DNA vaccine SL7207 (pVAX1-C), both in vitro and in vivo. The humoral and cellular immune responses in vivo were observed after oral immunization with the SL7207 (pVAX1-C) DNA vaccine. High titers of the specific antibody against the capsid protein and the neutralizing antibody against the DTMUV virus were both detected after inoculation. The ducks were efficiently protected from lethal DTMUV exposure by the SL7207 (pVAX1-C) vaccine in this experiment. Taken together, we demonstrated that the capsid protein of DTMUV possesses a strong immunogenicity against the DTMUV infection. Moreover, an oral delivery of the DNA vaccine SL7207 (pVAX1-C) utilizing *Salmonella* SL7207 was an efficient way to protect the ducks against DTMUV infection and provides an economic and fast vaccine delivery strategy for a large scale clinical use.

## 1. Introduction

The genus *Flavivirus* contains important anthropod-borne human pathogens, such as dengue virus (DENV), West Nile virus (WNV), yellow fever virus (YFV), Japanese encephalitis virus (JEV) and Zika virus (ZIKV) [1,2,3]. The genome of these flaviviruses consists of one single copy of positive-strand RNA that encodes three structural proteins: capsid, pre-membrane/membrane (prM/M) and envelope (E) proteins, as well as seven non-structural (NS) proteins: NS1, NS2A, NS2B, NS3, NS4A, NS4B, NS5 [4].

Duck tembusu virus (DTMUV) is a newly identified *Flavivirus* that was isolated from duck in China in 2010 [5] and causes severe symptoms, such as a decline in egg production, high fever, loss of appetite, retarded growth, or death. Since its outbreak, the spread of DTMUV has caused a huge economic loss in the poultry industry in China [5,6]. The spread and outbreak of DTMUV is hard to control because it is transmitted by arthropods, such as mosquitoes and also because of the wide range of potential hosts, such as chicken, goose, pigeon and sparrow [7,8,9]. In addition, it was reported that DTMUV has the potential to affect people [10]. Thus, the prevention and control of DTMUV infection and transmission in the poultry industry is urgently needed. Different kinds of DTMUV vaccines have been studied in previous studies but there is still room for further improvement. Although a live attenuated DTMUV vaccine by serial passaging in chicken embryo fibroblasts provides good immune responses, the virus could be measured in the vaccinated ducks’ tissues, which may pose the risk of reversible virulence [11]. To provide safer alternatives, a DTMUV beta-propiolactone-inactivated oil-emulsion vaccine and a purified DTMUV envelope protein containing liposome vaccine have been developed [12,13]. However, these two vaccines are impossible for large-scale inoculations in clinical practices, because of the high cost and intricate delivery. This has led to the exploration of new strategy in vaccine development, such as the oral DNA vaccine designed in this study [14].

Capsid proteins, the main structure proteins of flaviviruses, have been widely studied regarding to their function. The primary function of flaviviral capsid proteins is for genome packing [15]. Additionally, they can enhance the replication and translation of viral genomes during the production of infectious virions [16,17,18]. They also play a crucial role in modulating host cell signaling networks by affecting innate immunity, which benefits the replication of flaviviruses [19]. However, capsid proteins used as an antigen in vaccine development against flaviviruses is rarely mentioned or evaluated. Thus, based on attenuated *Salmonella* typhimurium which have been widely used to deliver heterologous antigens to the immune system [20], an oral DNA vaccine using a DTMUV capsid protein as the antigen was developed and evaluated against DTMUV infection in ducks.

## 2. Materials and Methods

### 2.1. Plasmid, Bacterial Strains, Virus and Ducks

Plasmid pVAX1 containing the eukaryotic expression promoter cytomegalovirus (CMV) and bovine growth hormone (BGH) poly A signal was purchased from Invitrogen (USA). SL7207, *Salmonella* typhimurium 2337-65 derivative hisG46, DEL407 [aroA::Tn10 (Tc^s^)], was kindly provided by Professor Kai Schulze of the Helmholtz Center for Infection Research (Germany). The DTMUV WR strain (GenBank: JX196334.1), isolated in Fujian, was generously provided by Professor Yu Huang from the Fujian Academy of Agricultural Sciences (China). This virus was propagated in the allantoic cavities of 9-day-old specific pathogen-free (SPF) embryonated duck eggs and stored in −80 °C until use. One-day-old shelducks were purchased from commercial duck farms in Ya’an, China. DTMUV-free ducks were confirmed by PCR. All animals were fed under standard conditions.

### 2.2. Construction of DNA Vaccine Plasmids

Total RNA of DTMUV was extracted from the allantoic fluid by Trizol (Invitrogen, Carlsbad, CA, USA) according to the manufacturer’s instructions and was reverse transcribed into cDNA. The capsid gene (GenBank: JX196334.1) was amplified with the primers listed in Table 1 from the cDNA template and cloned into the multiple cloning site of the pVAX1 vector using the *EcoRI* site (underlined) in the forward primer and the *XhoI* site (underlined) in the reverse primer. The resulted plasmid was named pVAX-C. pVAX-C and empty pVAX plasmids were transformed into SL7207 by electroporation [21] to generate an oral DNA vaccine SL7207 (pVAX-C) and SL7207 (pVAX).

### 2.3. Expression of the Capsid Protein from DNA Vaccine in Vitro

Plasmids pVAX-C and pVAX were prepared using the EndoFree Plasmid Kit (Tiangen, Beijing, China) and were transfected into COS7 cells using the *TransIntro*^TM^ EL Transfection Reagent (TransGen Biotech, Beijing, China) when the cells were growing at around 80% confluent in six-well plates. The expression of the capsid protein in the cells post 48 h transfection was confirmed by indirect immunofluorescence as described previously [21]. Briefly, transfected cells were washed with phosphate-buffered-saline (PH 7.2) (PBS), then fixed with 4% paraformaldehyde and permeabilized with 0.2% Triton X-100 in PBS and blocked with 5% BSA in PBS (BSA-PBS). After that, the cells were incubated with rabbit anti-capsid protein polyclonal antibody (prepared by our lab) as the primary antibody diluted 1:100 in BSA-PBS for 2 h, followed by incubation with the Alexa Fluor 488-conjugated goat anti-rabbit IgG (Thermo Fisher, Lafayette, CO, USA) as the secondary antibody was diluted 1:2000 in BSA-PBS for 2 h at room temperature. The cell nuclei were counterstained with 4′, 6-diamidino-2-phenylindole (DAPI) for 10 min at room temperature. The cells were examined by fluorescence microscopy (Nikon, Tokyo, Japan).

### 2.4. Sample Collection, Vaccination and Challenge Experiments

**Figure 1:**
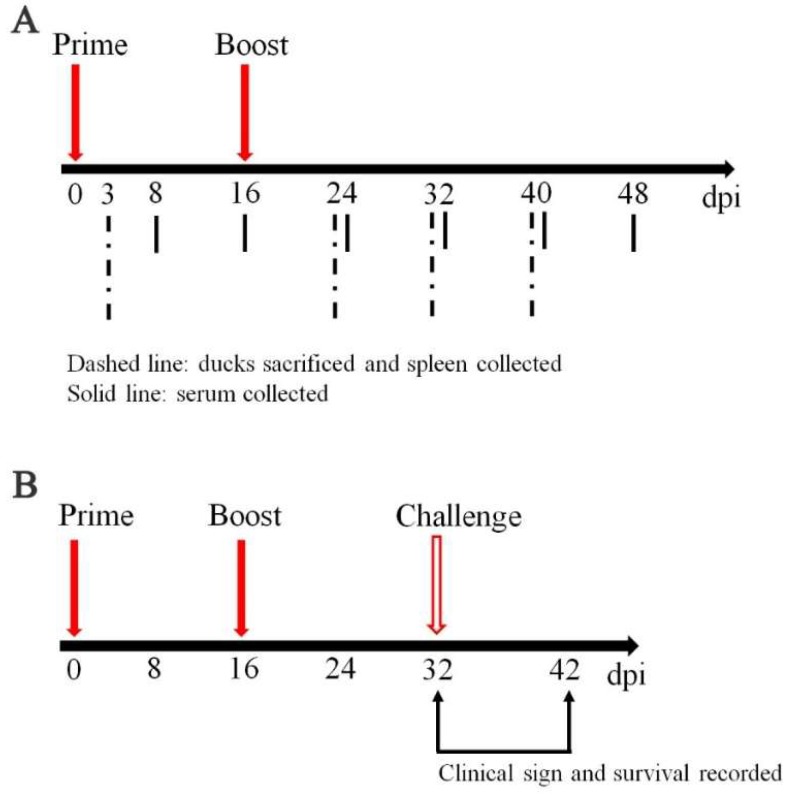
(**A**) Schedule of vaccination and sample collection. Ducks were orally vaccinated with 10^10^ CFU of SL7207 (pVAX-C) and SL7207 (pVAX) at 8-day-old and 24-day-old, respectively. Animals were sacrificed at 3, 24, 32 and 40 dpi to collect the spleen (*n* = 3 of each time point). Sera were collected at 8, 16, 24, 32, 40 and 48 dpi (*n* = 3 of each time point); (**B**) Schedule of challenge experiment. 10 ducks of each group were randomly selected at 32 dpi for the immune protection test and mortality was recorded for continuous 10 days after exposure of duck tembusu virus (DTMUV).

The animal experiments were approved by the Institutional Animal Care and the Use Committee of Sichuan Agricultural University (29 January 2014, Permit Number: SYXK (Chuan) 2014-187), China. 56 ducks at 7-day-old were randomly divided into 2 groups (28 ducks per group). Ducks from each group were vaccinated orally with 10^10^ colony-forming units (CFU) of SL7207 (pVAX-C) or SL7207 (pVAX) in 0.5 mL volume of PBS at 8-day-old and boosted at 24-day-old. At 3, 24, 32 and 40 days post first injection (dpi), the spleens of three ducks randomly selected from each group were collected and stored in −80 °C until use. At 8, 16, 24, 32, 40 and 48 dpi, sera were collected and stored in −80 °C before use (Figure 1A).

Sixteen days after the second immunization, 10 ducks from each group were randomly selected and challenged with 10^4.5^-fold 50% of embryo lethal death (ELD_50_) DTMUV per duck by intravenous injection. The clinical symptoms and death of those challenged ducks was checked and recorded for continuous 10 days afterwards (Figure 1B).

### 2.5. Expression of the Capsid Protein from DNA Vaccine in Vivo

The ducks in each group were euthanized and the spleens were collected at 3 dpi. The expression of capsid proteins in vivo was checked by immunohistochemistry, as described previously [22]. In brief, the spleen was fixed in 4% paraformaldehyde, embedded in paraffin and cut at 4 μm thickness (Leica RM2128, Wetzlar, Germany). The sections were dewaxed with xylene and re-hydrated through gradient ethanol and distilled water. Endogenous peroxidase activity was blocked by 0.3% hydrogen peroxide (H_2_O_2_). The sections were then soaked in citrate buffer solution (CBS, 0.01 M, PH 6.0) and submitted to antigen retrieval. Subsequently, the unspecific antigens were blocked by PBST (PBS containing 0.05% Tween-20, 0.01 M, PH 7.4) with 10% normal goat serum (S-PBST) for 1 h at 37 °C. Then the slides were incubated with a rabbit anti-capsid protein polyclonal antibody (prepared by our lab) as the primary antibody was diluted 1:200 in S-PBST and a horseradish peroxidase-conjugated goat anti-rabbit immunoglobulin G (IgG) (Transgen Biotech, China) was used as the secondary antibody was diluted 1:1000 in S-PBST. Next, the sections were stained by DAB (Solarbio, Beijing, China) and examined by microscope (Olympus BX43, Tokyo, Japan).

### 2.6. Quantitative RT-PCR

Total RNA of spleen collected from 24, 32 and 40 dpi was extracted using Trizol (Invitrogen, USA) according to the manufacturer’s manual. cDNA reverse transcribed from RNA was subjected to quantitative real-time polymerase chain reaction (quantitative RT-PCR) to check the expression level of IL-4 and IL-6 using the primers listed in Table 1. The procedure of quantitative RT-PCR was performed as previous work [23]. The results of the quantitative RT-PCR were analyzed by 2^−ΔΔ*C*t^ method and expressed as the mean ± standard deviation.

### 2.7. Enzyme-Linked Immunosorbent Assay (dELISA)

The presence of a specific anti-DTMUV capsid protein antibody from the serum in vaccinated ducks was examined by using indirect ELISA. 100 µL of purified capsid protein (1 µg/mL) as a capture molecule was incubated in 96-well ELISA plates at 4 °C overnight. The plates were washed three times with PBST and blocked by 1% bovine serum albumin (BSA) for 1 h at 37 °C. Serum samples diluted 1:400 in PBS were added into plates (100 µL per well) and incubated at 37 °C for 1 h. The plates were washed three times with PBST. After that, 100 µL of 1:2000 diluted horseradish peroxidase conjugated goat anti-bird IgY (Abcam, Cambridge, UK) was added to each well and incubated at 37 °C for 1 h. After washing three times, the plates were incubated with 3,3′,5,5′-tetramethy1 benzidine (TMB) as a substrate for 10 min. H_2_SO_4_ (2 mol/L) was added to each well to stop the reaction and the OD value at 450 nm was measured by using a Bio-Rad Model 860 Plate Reader (Bio-Rad, Hercules, CA, USA).

### 2.8. Neutralizing Assay

Neutralizing antibodies from sera collected at 8, 16, 24, 32, 40, 48 dpi was measured, as described previously [24]. Sera samples were inactivated at 56 °C for 30 min (*n* = 3) and diluted in serial twofold dilutions in MEM medium. Each sample was mixed with an equal volume of 100 TCID_50_ of DTMUV and incubated at 37 °C for 1 h. The mixture in each well was then replaced with BHK-21 cells and incubated and propagated for an additional 5 days. Titers of neutralizing antibodies were determined by monitoring the cytopathic effect (CPE). Each sample was repeated twice independently. Neutralizing activity was recorded until two out of three wells of infected cells showed no CPE.

## 3. Results

### 3.1. Expression of the Capsid Protein from the DNA Vaccine in Vitro and in Vivo

**Figure 2:**
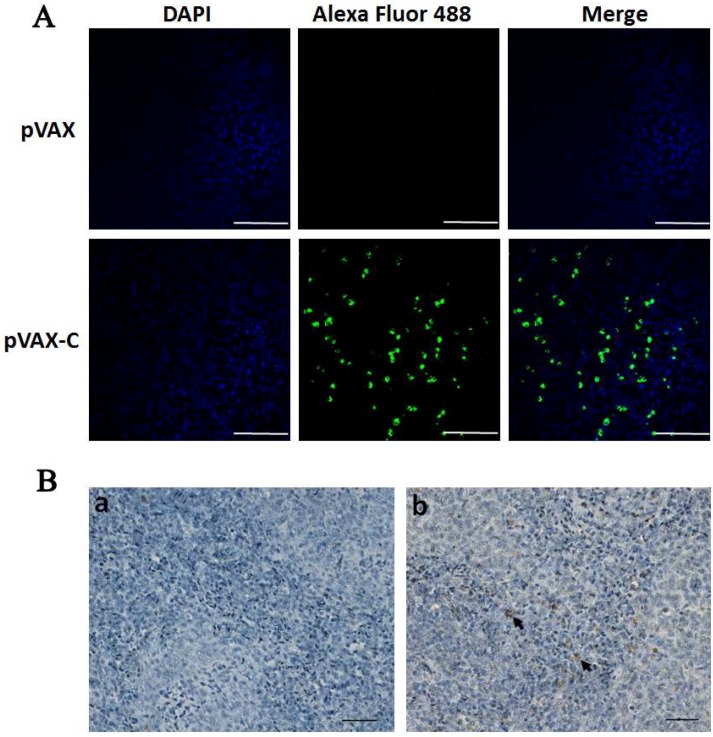
Expression of the capsid protein gene in vitro and in vivo. (**A**) COS7 cells were transfected with the DNA vaccine plasmids pVAX-C or pVAX. At 48 h post transfection, the cells were processed by indirect immunofluorescence assay using the anti-DTMUV-capsid protein rabbit polyclonal antibody as the first antibody and the Alexa Fluor 488-conjugated (green fluorescence) goat anti-rabbit as the secondary antibody. Scale bar 200 μm; (**B**) Spleens collected from the SL7207 (pVAX) (**a**) or the SL7207 (pVAX-C) vaccination group (**b**) at 3 dpi (*n* = 3). The capsid protein was detected by immunohistochemistry assay using the anti-DTMUV-capsid protein rabbit polyclonal antibody as the primary antibody and the horseradish peroxidase-conjugated goat anti-rabbit secondary antibody. The brown dots directed by the arrow indicated the capsid protein. Scale bar 50 μm.

Expression of the antigen capsid protein from the DNA vaccine plasmid pVAX-C was confirmed by indirect immunofluorescence assay in the transfected COS7 cells. After 48 h post transfection, specific green fluorescence which indicated the DTMUV capsid protein was observed in the cells transfected with pVAX-C, whereas no fluorescence was detected in cells transfected with empty vector pVAX (Figure 2A). The results indicated that the vaccine plasmid was successfully constructed and the capsid protein gene was successfully expressed from the DNA vaccine pVAX-C in vitro.

In order to investigate if the antigen was expressed in vivo via oral inoculation with the DNA vaccine SL7207 (pVAX-C), the capsid protein in the spleens collected at 3 dpi in the SL7207 (pVAX) or SL7207 (pVAX-C) vaccinated group was checked by using immunohistochemistry. As shown in Figure 2B, specific brown spots which represented the capsid protein antigen was observed on the slides from ducks orally inoculated with the DNA vaccine SL7207 (pVAX-C) (Figure 2Bb), whereas an absence of brown spots on the slides of the SL7207 (pVAX) group was observed (Figure 2Ba). These results indicated that the antigen capsid protein gene cloned in the pVAX eukaryotic vector was successfully expressed in vivo*.* Moreover, the DNA vaccine orally delivered by using attenuated *S.* typhimurium SL7207 was an efficient and successful inoculation method.

### 3.2. Cellular and Humoral Immune Responses in Ducks

**Figure 3:**
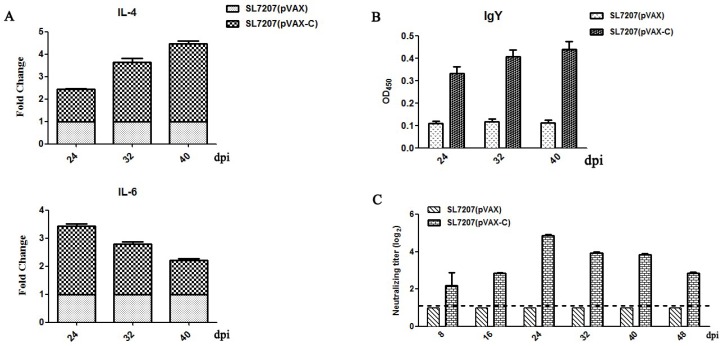
Immune responses stimulated by the vaccine. (**A**) Expression of IL-4 and IL-6 was measured by quantitative real-time polymerase chain reaction (RT-PCR) to evaluate the cellular immune responses. Data from RT-PCR was analyzed using 2^−ΔΔ*C*t^ method. The expression level of IL-4 and IL-6 in the SL7207 (pVAX-C) vaccinated group was presented as changed fold reference to those in the SL7207 (pVAX) group. Data are shown as the mean ± standard deviations (*n* = 3 of each time point); (**B**) The specific antibody IgY in the serum against the DTMUV capsid protein was detected by using indirect ELISA. The serum samples were incubated with the capsid proteins and detected by using the horseradish peroxidase conjugated goat anti-bird IgY. OD_450_ value of each well was measured. The titers of the specific antibody were presented as the means ± standard deviations (*n* = 3 of each time point); (**C**) Neutralizing antibodies against DTMUV in the serum was detected by neutralizing assay. The titers of neutralizing antibodies against DTMUV were detected and presented as the log_2_ changed folds (Y-axis) reference to the negative control group SL7207 (pVAX). Data are shown as the means ± standard deviations (*n* = 3 of each time point). The dash line indicates the lowest threshold value for positive reaction in the neutralizing assay. All data were graphed by GraphPad Prism v5.0 (La Jolla, CA, USA).

To evaluate the cellular immune response stimulated by our developed oral DNA vaccine, the expression of cytokine molecules IL-4 and IL-6 in the spleen was measured by quantitative RT-PCR. As shown in Figure 3A, the expression of IL-4 was up-regulated 2.5 to 4-fold from 24 to 40 dpi in the SL7207 (pVAX-C) immune group, compared with the SL7207 (pVAX) control group. In addition, the expression level of IL-6 was 2 to 3.5-fold higher in the SL7207 (pVAX-C) group than that in the SL7207 (pVAX) group, from 24 to 40 dpi. The expression of IL-4 was increased gradually from 24 to 40 dpi; in contrast, IL-6 presented the highest expression level at 24 dpi and subsequently dropped gradually. However, both IL-4 and IL-6 were overexpressed at least 2 folds in the SL7207 (pVAX-C) vaccine group during 24 to 40 dpi. These results indicated that the cellular immune response in vivo was efficiently induced by orally inoculating our developed DNA vaccine SL7207 (pVAX-C).

To check the specific humoral immune response induced by the oral SL7207 (pVAX-C) vaccine, antibody in the serum against the DTMUV capsid protein was analyzed by using indirect ELISA. As shown in Figure 3B, a high level of specific antibodies against the capsid protein in the oral vaccine SL7207 (pVAX-C) group was detected, which was 3 to 4-fold higher compared to the negative control SL7207 (pVAX) group. The specific antibody appeared quickly after the second inoculation and maintained at a high and stable level from 24 to 40 dpi. These results indicated that a specific antibody against the DTMUV capsid protein was successfully induced by our developed oral vaccine SL7207 (pVAX-C) in vivo*.*

### 3.3. Neutralizing Antibodies Responses

After the oral inoculation of SL7207 (pVAX-C) vaccine, neutralizing activity against the DTMUV virus from the serum was detected by using a neutralizing assay. As shown in Figure 3C, the titers of the neutralizing antibodies against DTMUV in the SL7207 (pVAX-C) vaccinated group were significantly higher, which showed 2 to 4.8 log_2_ folds changes, than that in the negative control group SL7207 (pVAX) at all time points. It reached to the peak at 24 dpi and maintained high levels during all the time points we tested. No neutralizing antibodies were detected in the negative control group SL7207 (pVAX). These results indicated that the oral SL7207 (pVAX-C) DNA vaccine efficiently induced the neutralizing antibodies against DTMUV in vivo*.*

### 3.4. Protection of Ducklings against DTMUV Challenge

**Figure 4:**
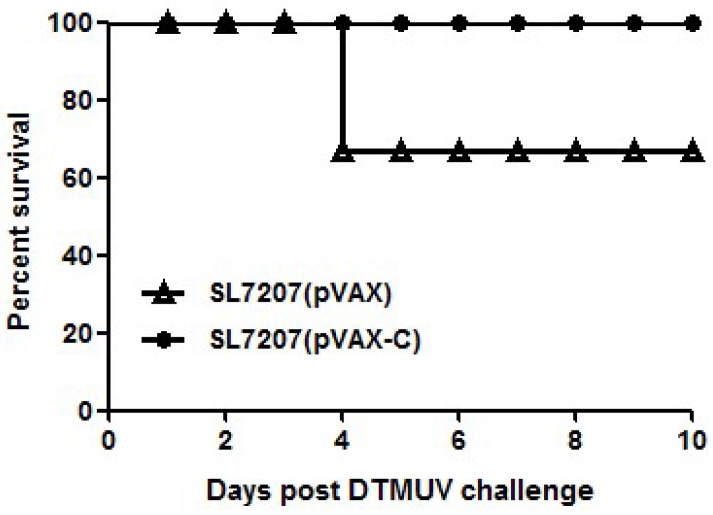
Survival post challenge with lethal doses of virulent DTMUV. The ducks (*n* = 10/group) were challenged with 10^4.5^-fold ELD_50_ DTMUV at 16 days after the second immunization. The death number of ducks was recorded for consecutive 10 days after virus intravenous injection and graphed by GraphPad Prism v5.0.

To verify the protective ability of our developed oral DNA vaccine against DTMUV, vaccinated animals were challenged with 10^4.5^-fold ELD_50_ DTMUV at 16 days after the second vaccination (Figure 1B). As shown in Figure 4, 30% ducks in the SL7207 (pVAX) vaccine group died; however, all ducks in the SL7207 (pVAX-C) vaccine group survived. Other than comparing the mortality between the vaccine and control groups, the clinical signs of the ducks were recorded. The typical clinical signs of the DTMUV infection, such as depression, inappetence and reluctance to move were observed in the surviving ducks in the non-vaccination group. However, these clinical signs were only slightly observed in a few of the ducks in the vaccinated group during 1 to 4 days post the DTMUV challenge and these ducks then soon recovered. These results indicate that the oral DNA vaccine SL7207 (pVAX-C) successfully protected the ducks against DTMUV infection.

## 4. Discussion

To the best of our knowledge, capsid protein of flaviviruses is not only as a genome guardian to capsulate viral genomes but also as a modulator to affect host cell signaling pathways [19]. Data from previous studies indicate that the capsid protein of hepatitis C virus (HCV) regulates the host innate immune responses by modulating the interferon regulatory factor (IRF), Jak-Stat and inducible nitric oxide synthase (iNOS) pathways to affect the persistence and pathogenesis of HCV [25]. Moreover, the semi-purified capsid proteins of DENV-2 immunized have the ability to protect mice against challenge with the homologous virus [26]. However, the immunogenic characteristics of DNA vaccine based on capsid protein of flaviviruses have not been explored. In this study, the DNA vaccine expressing the capsid gene of DTMUV delivered by attenuated *Salmonella* typhimurium SL7207 was designed. Our data showed that the oral DNA vaccine expressing the capsid protein could induce systemic immune responses against DTMUV. These results indicated that the capsid protein of DTMUV possesses strong antigenicity and immunogenicity and the SL7207 (pVAX-C) vaccine might be used as a candidate vaccine against DTMUV.

Eukaryotic expression plasmid pVAX1 has been extensively used as the backbone of the DNA vaccine and has helped virus genes express in eukaryotic cells [27,28]. Attenuated *Salmonella* typhimurium is a safe and efficient bacteria vector to carry a DNA vaccine [21,29]. Moreover, *S.* typhimurium as an enteropathogenic bacteria can deliver exogenous antigens to the immune system at a low cost [21,30]. Therefore, DNA vaccines encoding viral proteins carried by an attenuated *S.* typhimurium vehicle have been widely developed [31,32]. In this study, the capsid proteins were expressed efficiently in duck spleen cells when the ducks were orally inoculated with the vaccine SL7207 (pVAX-C) (Figure 2). This outcome indicated that the DNA vaccine pVAX-C carried by the attenuated *S.* typhimurium SL7207 was introduced successfully into body cells through oral immunization and the eukaryotic promoter CMV functioned to drive the expression of the capsid protein in eukaryotic cells after SL7207 degradation.

Usually, E protein of flaviviruses contains major protective epitopes [33,34]. Seven T cell epitopes were also identified in the capsid protein of DENV-4 [35]. This indicates that capsid protein of flaviviruses is also a target antigen of the anti-viral immune responses. This study has shown that the oral DNA vaccine SL7207 (pVAX-C) not only induced specific humoral antibody responses but also stimulated cellular mediated immune responses (Figure 3). The systemic immune responses were in agreement with previous results that the capsid protein of HCV induced strong humoral and cellular immune responses [36,37]. Molecule IL-6 is related to the development and differentiation of immune related cells, such as T helper cells and B cells [38,39]. In addition, IL-6 as a proinflammatory cytokine molecule has been proven to play an important role in regulating antiviral immunity [40]. An earlier and stronger expression of IL-6 has been found compared to other immune related genes including IFNγ, IL-1β, IL-2 and Cxcl8 in the DTMUV infected tissues; thus, IL-6 is deemed to be a key molecule in the cellular immune responses against DTMUV [23,41]. The high expression level of IL-6 was also observed post oral immunization of the DNA vaccine SL7207 (pVAX-C) (Figure 3A). The peak expression of IL-6 was earlier than IL-4 in the spleen after the vaccine inoculation. This may be because IL-4 is a key regulator in humoral immune responses [42]. To illustrate that, there was a positive relationship between the levels of IL-4 and the specific anti-capsid protein IgY antibody (Figure 3). Therefore, the capsid protein of DTMUV has an effective immunogenicity to induce systemic immune responses, as in the aforementioned findings.

Neutralizing antibodies have been regarded as the key factor for protection against flavivirus infection [43]. The high neutralizing activity and protective capacity can be observed in the present study. These may be related with several reasons. First, the *S.* typhimurium as an enteropathogenic carrier which delivers the pVAX-C DNA vaccine directly into the professional antigen presenting cells (APC) is feasible [44] and the bacterium containing lipopolysaccharide (LPS), which can activate Toll-like receptor 4, is also a potent adjuvant for enhancing immunogenicity of the DNA vaccine [45,46]. Second, it is a common knowledge that the capsid protein is necessary to guard the genetic materials of flavivirus to offspring [17,18]. Therefore, there were chances to against the DTMUV challenge through hindering the production of infectious virions. Third, the capsid dibasic-site, which is a cleavage region in the capsid protein, is important for virus growth [47]. As a result of blocking this site, the virus particle release from infected cells can be restricted [48]. Finally, increasing evidence indicates that capsid proteins can contact many host proteins to modulate the host immune signal in the cytoplasm and nuclei [19,49]. For instance, capsid proteins interact with importins and human Sec3 exocyst proteins to nullify the antiviral activity of the host [49,50]. Thus, the oral DNA vaccine expressing the capsid protein in ducks could produce neutralizing antibodies and form a protective ability against DTMUV infection.

Conclusively, the present study revealed that immunization with the oral DNA vaccine SL7207 (pVAX-C) induced strong humoral and cellular immune responses and provided an effective degree of protection against DTMUV infection. All of the data obtained here for the first time indicated that the capsid protein of DTMUV is a potentially protective antigen. Thus, capsid proteins are worthy to be further studied to develop efficient strategies to prevent DTMUV infection. In addition, an oral delivery of DNA vaccine SL7207 (pVAX1-C) utilizing *Salmonella* SL7207 provided an economic vaccine delivery strategy for a large scale clinical use to protect ducks against DTMUV infection.

## Figures and Tables

**Table 1:** Primers used in this study.

Name	Sequence (5′-3′)	Length of PCR Products (bp)	GenBank Number
C (Fw)	TACAGAATTCACTATGGCATCTAACAAAAAACCAGGAAGACCC	360	JX196334.1
C (Rev)	TACACTCGAGCTACCCAGCAACTATCGGGAGTAACATA		
Il-4 (Fw)	TCTATCAGAGAAAGACAACAC	157	XM_013104023.1
Il-4 (Rev)	GGTGACTATTTCTTTCAAGT		
Il-6 (Fw)	AAGTTGAGTCGCTGTGCT	120	JQ728554.1
Il-6 (Rev)	GCTTTGTGAGGAGGGATT		
GAPDH (Fw)	CAAGGCTGAGAATGGGAAAC	171	GU564233.1
GAPDH (Rev)	CTGCCCACTTGATGTTGC		
